# CO_2_ Fertilisation of Growth in Wild C_4_ Grasses may be Amplified by Upregulated Photosynthesis

**DOI:** 10.1111/pce.70785

**Published:** 2026-08-03

**Authors:** Edith J. Singini, Sally Archibald, Colin P. Osborne, Brad S. Ripley

**Affiliations:** ^1^ Botany Department Rhodes University Grahamstown South Africa; ^2^ School of Animal, Plant and Environmental Sciences University of the Witwatersrand Johannesburg South Africa; ^3^ Plants, Photosynthesis and Soil School of Biosciences, University of Sheffield Sheffield UK

**Keywords:** C_4_ photosynthesis, drought, elevated CO_2_, grassland ecology, photosynthetic upregulation, water use efficiency

## Abstract

Rising atmospheric CO_2_ is expected to have limited direct effects on C_4_ photosynthesis because the carbon‐concentrating mechanism maintains near‐saturating CO_2_ at Rubisco. Consequently, growth responses of C_4_ plants to elevated CO_2_ (eCO_2_) are generally attributed to indirect improvements in plant water relations. However, it remains unclear whether sustained exposure to eCO_2_ also induces changes in photosynthetic capacity that contribute directly to carbon gain. We examined the interactive effects of CO_2_ concentration and soil water availability on photosynthesis, biochemical capacity, and growth in three African C_4_ grasses grown under ambient (400 ppm) and elevated (550 ppm) CO_2_. Elevated CO_2_ increased net photosynthesis across species, with stronger responses under drought. Intrinsic water‐use efficiency increased primarily through enhanced assimilation, while stomatal responses were contingent on water availability rather than driven directly by elevated CO_2_. Importantly, eCO_2_ induced species‐ and context‐dependent increases in apparent photosynthetic capacity after prolonged treatment exposure. *Megathyrsus maximus* showed strong V_cmax_ increases under both water treatments and J_max_ upregulation under well‐watered conditions, *Heteropogon contortus* exhibited biochemical upregulation primarily under drought, and *Themeda triandra* showed little evidence of acclimation. Growth responses closely mirrored these physiological patterns, occurring only in species exhibiting coordinated biochemical adjustment. These results indicate that C_4_ responses to eCO_2_ cannot be explained solely by stomatal water saving and provide evidence that species‐specific changes in apparent photosynthetic capacity may contribute to sustained carbon gain, particularly under water limitation.

## Summary statement

Elevated CO_2_ enhanced photosynthesis across C_4_ grasses, but biomass gains were confined to *Heteropogon contortus* and *Megathyrsus maximus*, where responses were associated with species‐ and water‐dependent increases in apparent photosynthetic capacity rather than uniform reductions in stomatal conductance.

## 1 Introduction

Atmospheric CO_2_ concentration has increased from pre‐industrial values of approximately 280 µmol mol^−1^ to almost 430 µmol mol^−1^ today and is projected to continue rising throughout this century (IPCC 2021). In C_3_ plants, elevated CO_2_ (eCO_2_) typically enhances photosynthetic carbon assimilation by increasing substrate availability to Rubisco (Ribulose‐1,5‐bisphosphate carboxylase/oxygenase) and suppressing photorespiration, often resulting in increased growth unless constrained by sink strength or nutrient limitation (Ainsworth and Rogers 2007; Leakey et al. 2009). By contrast, C_4_ plants have historically been regarded as comparatively insensitive to eCO_2_ because their carbon‐concentrating mechanism (CCM) elevates CO_2_ partial pressure at the site of Rubisco, thereby maintaining near‐saturating conditions for carboxylation at current atmospheric concentrations (Collatz et al. 1992; Sage and Kubien 2007).

This biochemical framework has strongly shaped theoretical expectations and vegetation models, leading to the widespread assumption that increasing atmospheric CO_2_ will have minimal direct effects on C_4_ photosynthesis, particularly under well‐watered conditions (Osborne and Sack 2012; Tausz‐Posch et al. 2020). At the leaf scale, this assumption is supported by the shape of the C_4_ AC_i_ response curve: short‐term increases in atmospheric CO_2_ from current levels are predicted to shift the operating point only modestly along an already flattened portion of the curve, producing relatively small increases in light‐saturated CO_2_ assimilation (Wand et al. 1999; Sage and Kubien 2007; Pignon and Long 2020). Mechanistic modelling and chamber experiments suggest that doubling CO_2_ from ~350 to 700 µmol mol^−1^ increases C_4_ photosynthesis by approximately 10% under optimal conditions, substantially less than responses observed in C_3_ species (Collatz et al. 1992; Stokes et al. 2016; Scott and Smith 2022). Consistent with this prediction, FACE experiments on major C_4_ crops report no significant enhancement of net photosynthetic rate under well‐watered conditions, despite large increases in atmospheric CO_2_ (Leakey et al. 2009; Ainsworth and Long 2021).

However, the conclusion that C_4_ plants derive little benefit from rising CO_2_ has been over‐generalised by an emphasis on crops and conflating minimal direct biochemical stimulation with minimal ecological or growth responses. A synthesis for wild C_4_ grasses demonstrated that eCO_2_ increased net photosynthetic rate by 25% on average and whole‐plant growth by 33%, with intrinsic water‐use efficiency increasing by 72% (Wand et al. 1999). These responses were observed across multiple species and across varying experimental durations, challenging the assumption that C_4_ photosynthesis is uniformly CO_2_‐saturated at current atmospheric CO_2_ concentrations. Subsequent comparative analyses further indicate that C_4_ responses to CO_2_ vary widely among species and environments, reflecting differences in biochemical limitation, stomatal regulation, and resource availability (Ghannoum et al. 2000; Sage and Kubien 2007).

A key mechanism underlying C_4_ responses to eCO_2_ is indirect mediation through plant water relations. Across multiple studies, eCO_2_ consistently reduces stomatal conductance in C_4_ species by approximately 25–28%, thereby reducing transpiration and improving water‐use efficiency (Wand et al. 1999; Stokes et al. 2016; Quirk et al. 2019). These changes can improve plant water status and delay the onset of drought stress, thereby enhancing growth or productivity under water‐limited conditions. Consequently, CO_2_‐driven growth responses in C_4_ plants are often attributed less to direct increases in leaf‐level carbon assimilation and more to improvements in plant water relations (Ghannoum et al. 2000; Morgan et al. 2004).

Beyond water relations, recent biochemical analyses suggest that C_4_ photosynthetic capacity may not be optimally tuned to current atmospheric CO_2_ levels. Retrospective analysis of 49 C_4_ crop species showed that operating photosynthesis was more strongly limited by Rubisco carboxylation capacity (V_cmax_) than by PEP carboxylase capacity (V_pmax_), and that resource allocation between these components appeared optimised for pre‐industrial CO_2_ concentrations near 200 µmol mol^−1^ (Pignon and Long 2020). Modelling based on these data predicted that reallocating nitrogen from excess V_pmax_ toward V_cmax_ could increase C_4_ photosynthetic rate by approximately 12% at current CO_2_ levels (Pignon and Long 2020). Phylogenetic analysis similarly indicated that coordination of C_4_ biochemical traits remains optimised for low CO_2_ environments, with limited evolutionary adjustment to the rapid rise in atmospheric CO_2_ over recent decades (Edwards and Ogburn 2012; Pignon and Long 2020).

Together, the available evidence indicates that C_4_ responses to eCO_2_ are likely shaped by interactions among water availability, stomatal regulation, and adjustments in photosynthetic capacity. While reduced stomatal conductance and improved water‐use efficiency provide a well‐supported pathway for indirect growth enhancement under eCO_2_, it remains unresolved whether sustained exposure to eCO_2_ can also induce acclimatory changes in C_4_ photosynthetic capacity that contribute directly to increased carbon gain, particularly under water limitation. Addressing this gap requires explicit separation of stomatal and biochemical drivers of CO_2_ responsiveness and assessment of whether these responses are conserved or species‐specific.

In this study, we tested the hypothesis that growth stimulation under eCO_2_ in C_4_ grasses is not driven solely by stomatal water savings but is accompanied by acclimatory upregulation of photosynthetic capacity when carbon demand is sustained. Specifically, we hypothesised that: (1) prolonged growth at eCO_2_ increases photosynthetic capacity (V_cmax_, V_pmax_ and J_max_) in C_4_ species; (2) this photosynthetic upregulation is most pronounced under drought conditions, where reduced stomatal conductance at eCO_2_ improves plant water status and enables greater biochemical investment in photosynthetic capacity; and (3) the magnitude and direction of these responses differ among species, reflecting intrinsic differences in physiological plasticity. To test these hypotheses, we quantified AC_i_ response curves, stomatal conductance, and biomass responses of three ecologically important C_4_ grasses grown under ambient and eCO_2_ across contrasting soil water regimes, allowing direct evaluation of the mechanisms linking photosynthetic capacity, water relations, and growth under rising atmospheric CO_2_.

## 2 Materials and Methods

### 2.1 Species Selection

Three C_4_ grass species were selected based on their widespread occurrence in African savannas and grasslands, as well as their contrasting physiological responses to environmental change. *Heteropogon contortus* and *Themeda triandra* belong to the Tribe Andropogoneae and utilise the NADP‐malic enzyme (NADP‐ME) photosynthetic subtype (Ghannoum et al. 2001), whereas *Megathyrsus maximus* is a member of the Tribe Paniceae and employs the phosphoenolpyruvate carboxykinase (PCK) subtype (Voznesenskaya et al. 2006; Koteyeva et al. 2015). The main goal of the study was to distinguish between water‐saving and acclimatory upregulation as mechanisms of CO_2_ fertilisation, but the inclusion of three species from two photosynthetic subtypes also provided information on the generality of the results.

### 2.2 Experimental Design and Treatments

The experiment was conducted in open‐top chambers at the Rhodes University Elevated CO_2_ Facility in Makhanda, Eastern Cape, South Africa, a region with a warm temperate climate characterised by summer rainfall (long‐term mean of ~466 mm; CHIRPS) and seasonal variability in temperature (Supporting Information Figure S1). The experiment employed a split‐plot factorial design to examine the interactive effects of atmospheric CO_2_ concentration and soil water availability on grass physiological performance and growth. Atmospheric CO_2_ was manipulated at the whole‐plot (chamber) level, while water availability was applied at the subplot (pot) level within each chamber. Sixteen open‐top chambers (OTCs; 3 m diameter, 2.8 m height) were randomly assigned to either a controlled ambient/reference CO_2_ (aCO_2_; 400 ppm, *n* = 8 chambers) or eCO_2_ (550 ppm, *n* = 8 chambers). Chamber CO_2_ concentrations were monitored continuously, and daily mean concentrations for chambers assigned to each CO_2_ treatment are shown in Supporting Information Figure S1.

Each OTC contained 12 pots, each holding a single grass seedling, with four individuals per species per chamber. Within chambers, pots were randomly allocated to either a well‐watered or dry treatment, resulting in two individuals per species per water treatment per chamber. Across chambers (*n* = 8 per CO_2_ level), this yielded up to 16 individuals per species per CO_2_ × water treatment combination. It was not possible to take physiological measurements on all 192 pots, so for physiological and growth analyses, a random subset of more than seven individuals per species per CO_2_ × water treatment combination was analysed.

Seedlings were grown in 4 L cylindrical pots (approximately 11.3 cm in diameter and 40 cm in height) filled with homogenised savanna soil (Lombard's Post, Bathurst, Eastern Cape), with no fertilisers added during the experiment. The soil was sandy and nutrient‐poor, with baseline properties consistent with low‐fertility savanna soils. Prior to the experiment, the soil had a sand content of 90.2 ± 1.15%, silt content of 9.5 ± 1.10% and clay content of 0.23 ± 0.044%. Soil pH was 6.3 ± 0.18, carbon concentration was 0.72 ± 0.019%, nitrogen concentration was 0.07 ± 0.001%, bulk density was 1.46 ± 0.024 g cm^‐3^ (Hawkins et al. 2026), and the gravimetric water‐holding capacity was approximately 45% on a dry‐soil basis. These properties indicate that plants were grown in a sandy, low‐carbon and low‐nitrogen savanna soil. Because the same homogenised soil was used across all treatments, soil nutrient status was not confounded with CO_2_ or water treatment, although nutrient limitation may have constrained the absolute magnitude of plant responses to eCO_2_. Seeds were germinated in October 2021 in vermiculite and transplanted into pots in December 2021. All pots were placed near the centre of each OTC to minimise edge effects associated with shading and chamber microclimate heterogeneity. Seedling allocation to pots, water treatments, and chambers was fully randomised at transplanting to avoid systematic bias and ensure even representation across the experimental design.

Atmospheric CO_2_ concentrations within each OTC were continuously monitored using open‐path infra‐red gas analysers (GMP343, Vaisala, Finland) and regulated via a proportional–integral–derivative (PID) control system connected to proportional valves, ensuring stable target concentrations and uniform air mixing through a perforated diffuser and canopy‐level fan. Daytime CO_2_ concentrations averaged 398 ppm (central 90% range: 383–411 ppm) in aCO_2_ chambers and 545 ppm (central 90% range: 496–588 ppm; Hawkins et al. 2026) in eCO_2_ chambers, representing an enrichment of approximately 150 ppm consistent with mid‐21st‐century projections (IPCC 2021). Although current atmospheric CO_2_ concentrations are now closer to 420–430 ppm, the experiment was initiated using the established facility target of 400 ppm as the ambient reference treatment. The purpose of the treatment design was therefore to impose a controlled enrichment of approximately 150 ppm above the reference concentration, rather than to reproduce the exact atmospheric CO_2_ concentration.

Water availability was manipulated at the pot level. Well‐watered plants were maintained close to field capacity through regular irrigation, whereas dry‐treatment plants received no routine watering. To prevent irreversible damage or mortality during extended dry periods, minimal supplemental watering was applied only when severe wilting was observed. Water treatments commenced in January 2022, once seedlings were fully established. Total rainfall over the 12‐month period was 301 mm, below the site's long‐term mean (~466 mm, Climate Hazards Group infra‐red Precipitation with Station data). Because rainfall affected all chambers uniformly, it was not included explicitly as a model covariate; instead, its effects were captured through direct measurements of soil volumetric water content (VWC; Supporting Information Figure S2).

Microclimatic conditions within OTCs were monitored using five sensors that recorded air temperature and relative humidity at 10‐min intervals (HygroVUE, Campbell Scientific, Somerset West, RSA), allowing calculation of vapour pressure deficit (VPD; Supporting Information Figure S1). Soil moisture was monitored continuously using 5TM sensors (Campbell Scientific) installed in four randomly selected pots per chamber, with two sensors assigned to each water treatment. VWC was logged every 10 min throughout the experiment. Within each chamber, sensor values were averaged by water treatment to generate one VWC time series per chamber × water treatment combination (Supporting Information Figure S2). For physiological analyses, each gas‐exchange measurement was paired with the instantaneous treatment‐level VWC recorded for the corresponding chamber and water treatment. For growth analyses, time‐weighted mean VWC values were calculated over the treatment period to represent cumulative soil water availability experienced by each plant. Within each chamber, sensor values were averaged by water treatment, yielding one VWC time series per chamber × treatment combination (Supporting Information Figure S2). Relationships between VWC and soil water potential (Ψ_soil_) were interpreted with reference to previously established calibration curves obtained in comparable soils using MP‐6 soil water potential sensors (Delta‐T Devices; Ripley et al. 2022; Supporting Information Figure S3). To quantify the duration of low soil water availability, daily mean VWC values were calculated for each water × CO_2_ treatment combination across the 334‐day monitoring period. We then calculated the number and percentage of days below selected VWC thresholds of 18%, 15%, 12% and 10%. These thresholds were used descriptively to summarise treatment separation and drought severity, and were interpreted with reference to the soil water retention relationship shown in Supporting Information Figure S3. Although direct plant water status measurements were not made, this approach allowed us to quantify the duration of soil water limitation experienced by plants in each treatment.

### 2.3 Measurements

Physiological measurements were conducted on new growth during the austral spring between 20 September and 17 October 2022, approximately 10 months after transplanting. Measurements followed a block‐randomised daily schedule that alternated chambers, water treatments, and species to ensure balanced sampling across dates and times of day (09:00–15:00). For a subset of plants from each species and treatment combination (*n* = 7–12 plants per species × treatment combination; exact n values are provided in Supporting Information Table S2), we quantified leaf gas exchange responses and total biomass. Gas‐exchange measurements were performed using a portable photosynthesis system (LI‐6800, LI‐COR Biosciences). For each selected plant, AC_i_ response curves were measured, capturing net CO_2_ assimilation rate (A), intercellular CO_2_ concentration (C_i_), and stomatal conductance (g_sw_) across a range of soil moisture conditions (VWC approximately 5%–40%) reflecting the experimental treatments.

AC_i_ curves were measured during a single late‐season measurement campaign rather than repeatedly through time. This timing was selected to assess treatment‐associated physiological differences after prolonged exposure to the CO_2_ and water treatments. Because AC_i_ curves are time‐intensive, measurements were conducted using a block‐randomised daily schedule that alternated chambers, water treatments and species to minimise confounding between treatment and measurement date or time of day. Consequently, the AC_i_ data provide a late‐season assessment of apparent photosynthetic capacity after long‐term treatment exposure, but do not resolve seasonal variation in photosynthetic acclimation.

AC_i_ curves were generated using the automated CO_2_ response protocol of the LI‐6800 system, which sequentially adjusted reference CO_2_ concentrations and logged data only once predefined stability criteria were met. This automated approach ensured consistent equilibration across measurements, reduced observer bias, and improved the reliability of curve fitting. For ambient‐grown plants, the CO_2_ sequence was 400, 300, 200, 100, 50, 400, 600, 800, 1000 and 1200 ppm, while 550 ppm replaced 400 ppm for eCO_2_‐grown plants. This range captured PEPC‐, Rubisco‐ and RuBP‐regeneration‐limited phases of photosynthesis, with maximum CO_2_ concentrations sufficient to achieve assimilation saturation across all species. For subsequent analyses, values of A and g_sw_ were extracted from AC_i_ curves at the growth CO_2_ concentration of each treatment (400 µmol mol^−1^ for aCO_2_–grown plants and 550 µmol mol^−1^ for eCO_2_–grown plants). These extracted values were used to compare photosynthesis, stomatal conductance and intrinsic water‐use efficiency among treatments. They should be interpreted as gas‐exchange rates at growth CO_2_ under standardised cuvette conditions, rather than as independent spot measurements under chamber growth conditions. No repeated spot gas‐exchange measurements were made under the naturally varying light, temperature and VPD conditions within the OTCs. Intrinsic water‐use efficiency (iWUE) was calculated as the ratio of A to g_sw_ (A/g_sw_) at these respective CO_2_ concentrations.

Measurements were conducted on fully expanded healthy leaves under standardised conditions: photosynthetic photon flux density of 1500 µmol m^−2^ s^−1^, leaf temperature maintained at 25°C, fan speed of 10,000 rpm, and cuvette VPD maintained below 1.6 kPa. These standardised conditions were used to minimise short‐term environmental variation and allow comparison of apparent photosynthetic capacity across species, CO_2_ and water treatments. Leaf area was quantified using digital calipers. Photosynthetic parameters were estimated in R using the ‘C4Estimation’ fitting routines associated with Zhou et al. (2019), which implement C_4_ biochemical AC_i_ curve‐fitting procedures based on established C_4_ photosynthesis models (von Caemmerer and Furbank 1999; von Caemmerer 2000; von Caemmerer 2021). The fitting procedure estimates apparent maximum rate of PEPC (Phosphoenolpyruvate carboxylase) carboxylation (V_pmax_), the maximum rate of Rubisco carboxylation (V_cmax_), and the maximum electron transport rate (J_max_). These parameters were interpreted as apparent biochemical capacities because fits were based on intercellular CO_2_ (C_i_), and mesophyll conductance and bundle sheath conductance were not independently measured.

The ‘C4Estimation’ package uses non‐linear curve fitting to estimate C_4_ photosynthetic parameters from gas‐exchange measurements (Zhou et al. 2019). In our implementation, each replicate AC_i_ curve was fitted independently, and biologically implausible fits were excluded before statistical analysis. Because C_4_ AC_i_ fitting is sensitive to assumptions about mesophyll conductance, bundle sheath conductance and electron transport partitioning, we interpret treatment effects on V_cmax_, V_pmax_ and J_max_ as relative differences in apparent photosynthetic capacity rather than absolute biochemical rates. Stomatal and non‐stomatal limitation indices shown in Supporting Information Figure S4 were calculated from predicted assimilation values derived from fitted AC_i_ curves measured under standardised cuvette conditions; full equations and calculation details are provided in Supporting Information Methods S1.

Temperature‐dependent parameters, including the CO_2_ compensation point in the absence of dark respiration (Γ*) and the Michaelis–Menten constants for Rubisco (K_c_ and K_o_), were calculated from measured leaf temperature using standard parameterisations implemented in the ‘C4Estimation’ package (Zhou et al. 2019). The oxygen concentration was fixed at 21 kPa. PEP regeneration was not independently estimated; instead, it was represented by assuming a fixed fraction of total electron transport allocated to PEP regeneration (x = 0.4). Bundle sheath conductance to CO_2_ (g_bs_) was also not estimated from the data and was assigned using the package's temperature‐dependent parameterisation. For each C_i_ step, net assimilation was calculated as the minimum of PEPC‐limited, Rubisco‐limited, and RuBP‐regeneration‐limited rates. Mean predicted assimilation rates and associated standard errors were calculated across C_i_ values to summarise each plant's photosynthetic response profile.

Species‐level responses to eCO_2_ were classified into three categories following Singini et al. (2025): upregulation, limited or no upregulation, and downregulation. Upregulation was inferred when eCO_2_ produced (i) a significant positive effect on V_cmax_, V_pmax_, or J_max_ in mixed‐effects models, either as a positive CO_2_ main effect or as a significant positive elevated‐versus‐ambient CO_2_ contrast within a species × water treatment combination, and (ii) a clear upward shift in photosynthesis at growth CO_2_ supported by model estimates or post‐hoc contrasts. Limited or no upregulation was assigned when these criteria were not met, while downregulation was defined by significant negative parameter responses accompanied by reduced photosynthesis under eCO_2_.

Following completion of physiological measurements, plants were harvested destructively in November 2022 (n = 7–12 plants per species × treatment combination; exact n values are provided in Supporting Information Table S2). Total biomass was calculated as the sum of above‐ and below‐ground biomass. Above‐ground biomass was harvested by clipping plants at the soil surface, while below‐ground biomass was obtained by excavating the roots and washing them to remove adhering soil. Both biomass fractions were oven‐dried at 70°C for at least 72 h until a constant mass was reached and subsequently weighed to the nearest 0.01 g.

### 2.4 Data Analysis

All statistical analyses were conducted in R version 4.4.2 (R core team 2024). Prior to formal analysis, response variables were visually assessed for normality and homoscedasticity using Q–Q plots and residual‐versus‐fitted value plots. Where required, variables were transformed to improve model fit and stabilise variance. All analyses were performed per trait, with missing observations excluded at the model level. To examine the effects of atmospheric CO_2_ concentration and water availability on physiological and growth responses, linear mixed‐effects models were fitted using the ‘lme4’ package (Bates et al. 2015). Separate models were fitted for each response variable, including A, g_sw_, iWUE, total biomass, V_pmax_, V_cmax_, and J_max_. In all models, species identity, water treatment (watered vs droughted), CO_2_ treatment (ambient 400 ppm vs elevated 550 ppm), and their two‐ and three‐way interactions were specified as fixed effects. Chamber number and measurement date were included as random intercepts to account for non‐independence arising from shared growth conditions and temporal sampling effects. Models were fitted using restricted maximum likelihood (REML). Inference on fixed effects was based on Type III tests obtained using the ‘lmerTest’ package (Kuznetsova et al. 2017), with sum‐to‐zero contrasts applied to facilitate interpretation in the presence of interactions. The denominator degrees of freedom were estimated using Satterthwaite's approximation. Interaction terms were initially included in all models and subsequently removed when non‐significant; the final model structure reported was obtained by refitting the models. Statistical significance was assessed at *p* < 0.05. To quantify the magnitude and direction of CO_2_ effects within species and water treatments, estimated marginal means were calculated using the ‘emmeans’ package (Lenth 2020). Species‐specific CO_2_ effects were expressed as the difference between elevated and ambient CO_2_ (550 − 400 ppm) within each species × water treatment combination. *p*‐values for these contrasts were adjusted using the Sidak method to control for multiple comparisons. For each contrast, estimated differences (ΔeCO_2_), 95% confidence intervals, t‐statistics with Satterthwaite‐adjusted degrees of freedom, and adjusted *p*‐values are reported.

## 3 Results

Plants experienced a wide and continuous range of soil volumetric water content (VWC), spanning approximately 5%–40% over the experimental period (Supporting Information Figure S2). Because the experimental soil was sandy and low in organic matter, the soil water retention relationship indicated a steep decline in soil water potential as VWC decreased across the dry range (Supporting Information Figure S3). Based on this relationship, watered plants remained above the VWC threshold corresponding to approximately −0.45 MPa soil water potential throughout the experiment. In contrast, droughted plants remained below the threshold corresponding to approximately −1.25 MPa for the entire 334‐day monitoring period and below the threshold corresponding to approximately −2.38 MPa for more than 90% of the period (Supporting Information Table S1). Thus, although plant water potential was not measured directly, the continuous VWC records and soil water retention relationship indicate that droughted plants experienced prolonged low soil water availability, while watered plants remained in a substantially wetter range.

Across all species, net photosynthetic rate (A) responded strongly to atmospheric CO_2_ concentration, water availability, and species identity (Table 1). AC_i_ response curves showed a consistent upward shift under eCO_2_ relative to aCO_2_ in all three species under well‐watered conditions (Figure 1). These shifts were evident across a broad range of intercellular CO_2_ concentrations and were not confined to the operating point at growth CO_2_, indicating changes beyond instantaneous CO_2_ stimulation. Under drought, photosynthetic rates declined across species; however, eCO_2_ partially offset drought‐induced reductions, with particularly strong responses in *M. maximus* and *H. contortus* (Figure 1). Statistical analysis of photosynthesis at growth CO_2_ confirmed significant main effects of CO_2_, water treatment, and species, as well as significant two‐ and three‐way interactions among these factors (Table 1). Species‐specific contrasts showed that eCO_2_ significantly increased photosynthesis in all species under drought (ΔeCO_2_ = + 3.02 to + 10.17 µmol m^−2^ s^−1^; all *p* < 0.001), whereas under well‐watered conditions, CO_2_ stimulation was greatest in *H. contortus* and *M. maximus* and smaller in *T. triandra* (Table 2; Figure 2).

**Table 1:** Type III ANOVA results from linear mixed‐effects models evaluating the effects of species identity, water treatment, atmospheric CO_2_ concentration, and their interactions on photosynthetic rate (A), stomatal conductance (g_sw_), intrinsic water‐use efficiency (iWUE), total biomass, maximum rate of Rubisco carboxylation (V_cmax_), maximum rate of PEPC carboxylation (V_pmax_), and maximum electron transport capacity (J_max_). All interaction terms were initially included in the models and subsequently removed when non‐significant, after which the models were refitted. Degrees of freedom were estimated using Satterthwaite's approximation.

Trait	Effect	Df_1_	Df_2_	*F*	*p*
A	CO_2_	1	9.87	10.23	**0.010**
	Water	1	60.54	145.79	**< 0.001**
	Species	2	76.97	24.41	**< 0.001**
	Species × CO_2_	2	77.23	4.78	**0.011**
V_cmax_	CO_2_	1	42.05	33.77	**< 0.001**
	Water	1	7.93	7.43	**0.026**
	Species	2	63.32	1.41	0.252
	Species × CO_2_	2	62.81	9.2	**< 0.001**
V_pmax_	CO_2_	1	55.54	29.5	**< 0.001**
	Water	1	8.85	0.98	0.348
	Species	2	72.63	0.62	0.541
	Species × Water	2	72.66	3.9	**0.025**
J_max_	CO_2_	1	51.51	26.76	**< 0.001**
	Water	1	10.54	0.12	0.736
	Species	2	64.27	5.39	**0.007**
	Species × CO_2_	2	66.71	3.1	0.052
	Species × Water × CO_2_	2	67.81	8.92	**< 0.001**
g_sw_	CO_2_	1	57.07	3.25	0.077
	Water	1	10.29	0.09	0.770
	Species	2	72.41	1.92	0.154
	Water x CO_2_	1	60.04	4.04	**0.049**
iWUE	CO_2_	1	58.29	28.8	**< 0.001**
	Water	1	9.96	3.24	0.102
	Species	2	72.54	5.25	**0.007**
Total biomass	CO_2_	1	47.85	15.87	**< 0.001**
	Water	1	11.59	0.14	0.715
	Species	2	62.73	6.69	**0.002**
	Species × CO_2_	2	48.46	5.16	**0.009**
	Species × Water	2	62.7	3.73	**0.029**

**Figure 1:**
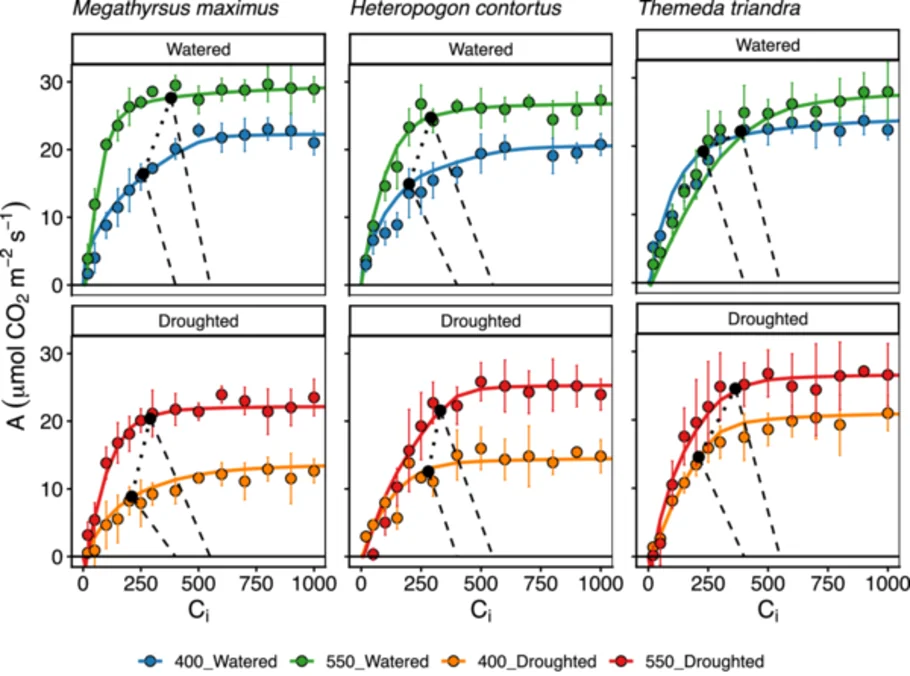
Species‐specific photosynthetic responses to internal CO_2_ concentration (C_i_) under ambient (400 ppm) and elevated (550 ppm) atmospheric CO_2_ across contrasting water treatments. AC_i_ response curves are shown for *Megathyrsus maximus*, *Heteropogon contortus*, and *Themeda triandra* under watered (top row) and droughted (bottom row) conditions. Mean points (±SE) represent measured gas‐exchange observations, while solid lines show fitted AC_i_ curves using the ‘C4Estimation’ package in R (Zhou et al. 2019). Dashed lines represent stomatal limitation at the growth CO_2_ concentration (Supporting Information Figure S4), calculated for each curve as the difference between assimilation at the growth intercellular CO_2_ (C_i_) and assimilation at the corresponding growth atmospheric CO_2_ (C_a_). Differences between ambient and elevated CO_2_ highlight species‐ and water‐dependent variation in photosynthetic acclimation (*n* = 7–12 plants per species × treatment combination; exact n values are provided in Supporting Information Table S2).

**Table 2:** Model estimated marginal mean contrasts comparing elevated (550 ppm) versus ambient (400 ppm) atmospheric CO_2_ concentrations within each species × water treatment combination. Values are expressed as ΔeCO_2_ (550 − 400) with corresponding 95% confidence intervals. Traits analysed include net photosynthetic rate (A), maximum rate of Rubisco carboxylation (V_cmax_), maximum rate of PEPC carboxylation (V_pmax_), maximum electron transport capacity (J_max_), intrinsic water‐use efficiency (iWUE), stomatal conductance (g_sw_), and total biomass (TB) for *Heteropogon contortus*, *Megathyrsus maximus*, and *Themeda triandra*. Reported *t*‐statistics (with Satterthwaite‐approximated degrees of freedom) and associated *p*‐values indicate the significance of CO_2_ effects within each treatment combination. Statistically significant effects (*p* < 0.05) are bolded, whereas non‐significant effects are shown in plain text.

Species	Water	Variables	ΔeCO_2_	95% CI	*t* (df)	*p*
*H. contortus*	Watered	A	8.8	5.74–12.02	5.63 (75.8)	**< 0.001**
	Droughted		10.17	7.35–12.99	7.20 (70.6)	**< 0.001**
*M. maximus*	Watered		6.75	3.78–9.72	4.52 (76.8)	**< 0.001**
	Droughted		7.03	3.99–10.07	4.60 (74)	**< 0.001**
*T. triandra*	Watered		3.02	0.36–5.68	2.26 (77)	**0.03**
	Droughted		6.29	3.46–9.11	4.43 (72.4)	**< 0.001**
*H. contortus*	Watered	g_sw_	−0.06	−0.16–0.04	−1.15 (67.9)	0.25
	Droughted		−0.06	−0.15–0.03	−1.3 (61.6)	0.2
*M. maximus*	Watered		−0.03	−0.13 − 0.07	−0.66 (65.9)	0.51
	Droughted		0.07	−0.03 − 0.17	1.38 (64.9)	0.17
*T. triandra*	Watered		−0.02	−0.1 − 0.07	−0.37 (65.8)	0.71
	Droughted		−0.07	−0.17 − 0.02	−1.49 (65.1)	0.14
*H. contortus*	Watered	V_cmax_	31.3	1.97 – 60.62	2.13 (61.5)	0.062
	Droughted		24.5	−0.37 – 49.33	1.97 (59)	0.057
*M. maximus*	Watered		71.9	43.68 – 100.10	5.09 (62.2)	**< 0.001**
	Droughted		61.9	34.38 – 89.4	4.5 (60.1)	**< 0.001**
*T. triandra*	Watered		−3.03	−30.31 – 24.24	−0.22 (60.4)	0.668
	Droughted		3.60	−21.53 – 28.73	0.29 (59.4)	0.785
*H. contortus*	Watered	V_pmax_	24.65	−32.45 − 77.74	0.82 (64.5)	0.33
	Droughted		42.42	−5.87 − 90.71	1.76 (59.3)	0.07
*M. maximus*	Watered		106.94	50.11 − 163.77	3.76 (63.4)	**< 0.001**
	Droughted		48.67	−3.44 − 100.77	1.87 (62.4)	0.05
*T. triandra*	Watered		22.27	−37.09 − 81.63	0.75 (64.2)	0.38
	Droughted		49.05	0.5 − 97.61	2.02 (61.3)	**0.04**
*H. contortus*	Watered	J_max_	41.07	−74.12 – 156.26	0.71 (70.5)	0.525
	Droughted		222.63	122.48 – 322.79	4.44 (62.2)	**< 0.001**
*M. maximus*	Watered		259.06	149.32 – 368.8	4.71 (70.7)	**< 0.001**
	Droughted		−24.18	−133.74 – 85.39	−0.44 (65.6)	0.659
*T. triandra*	Watered		26.09	−82.35 – 134.53	0.48 (68.2)	0.581
	Droughted		45.73	−55.18 – 146.64	0.91 (63.6)	0.392
*H. contortus*	Watered	iWUE	82.1	−2.53 – 166.73	2.03 (71.8)	**0.046**
	Droughted		90.18	13.99 – 166.37	2.36 (69.4)	**0.021**
*M. maximus*	Watered		41.13	−38.71 – 120.97	1.03 (74.5)	0.31
	Droughted		98.29	–16.23 – 180.36	2.38 (72.5)	**0.02**
*T. triandra*	Watered		25.34	−47.24 – 97.93	0.7 (74.9)	0.49
	Droughted		81.38	−2.23 – 160.52	2.05 (72.9)	**0.044**
*H. contortus*	Watered	TB	71.95	25.75 – 118.14	3.1 (73.8)	**0.003**
	Droughted		71.22	29.58 – 112.86	3.41 (71.4)	**0.001**
*M. maximus*	Watered		68.46	24.84 – 112.08	3.13 (76.5)	**0.003**
	Droughted		56.7	11.85 – 101.54	2.52 (74.5)	**0.01**
*T. triandra*	Watered		27.8	–11.36 – 66.91	1.41 (76.5)	0.16
	Droughted		−30.25	–72.07 – 11.56	−1.44 (73.4)	0.15

**Figure 2:**
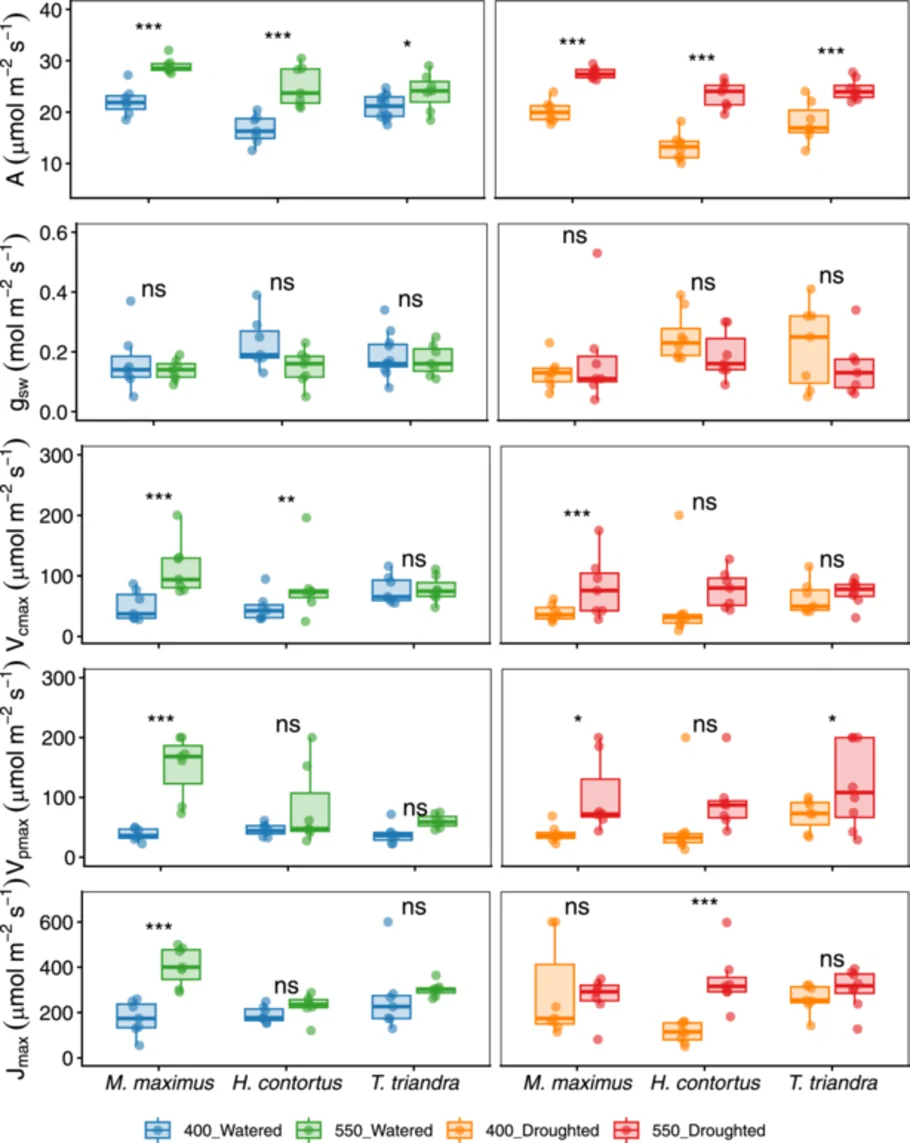
Photosynthetic and biochemical responses of three grass species (*Heteropogon contortus*, *Megathyrsus maximus*, and *Themeda triandra*) to atmospheric CO_2_ concentration and water availability. Panels show net photosynthetic rate (A), stomatal conductance (g_sw_), maximum rate of Rubisco carboxylation (V_cmax_), maximum rate of PEPC carboxylation (V_pmax_) and maximum electron transport rate (J_max_) measured under watered (left) and droughted (right) conditions at ambient (400 ppm) and elevated (550 ppm) CO_2_. Bars represent means ± SE (*n* = 7–12 plants per species × treatment combination; exact n values are provided in Supporting Information Table S2). Asterisks indicate significant differences between CO_2_ treatments within each species and water treatment. [Color figure can be viewed at wileyonlinelibrary.com]

The CO_2_‐induced shifts in AC_i_ curves reflected changes in curve shape and asymptote rather than simple displacement along a fixed response, providing evidence of treatment‐associated differences in apparent photosynthetic capacity at the time of measurement. While the quality of individual curve fits varied among replicates, fitted curves generally captured the observed response across the C_i_ range, and patterns of parameter variation were consistent at the species level (Figure 1). Consistent with this, biochemical parameters derived from curve fitting revealed species‐specific patterns of upregulation under eCO_2_. V_cmax_ differed significantly among species and showed a strong species × CO_2_ interaction (Table 1). eCO_2_ caused an increase of V_cmax_ in *M. maximus* under both well‐watered and droughted conditions (ΔeCO_2_ = +71.9 and +60.64 µmol m^−2^ s^−1^, respectively; both *p* < 0.001). In *H. contortus*, V_cmax_ responses to eCO_2_ were positive but marginal under both water treatments (*p* ≈ 0.06), indicating limited upregulation, whereas *T. triandra* showed no evidence of V_cmax_ upregulation under either water regime (Table 2; Figure 2, Supporting Information Table S2).

V_pmax_ showed an overall positive response to eCO_2_; however, responses were less consistent than those of V_cmax_ and were not strongly structured by water availability (Table 1). Species‐specific contrasts revealed that *M. maximus* exhibited a marked increase in V_pmax_ under well‐watered conditions (ΔeCO_2_ = +106.94 µmol m^−2^ s^−1^; Table 2), whereas V_pmax_ responses under drought were weaker and less consistent (Table 2; Figure 2, Supporting Information Table S2). In *H. contortus*, V_pmax_ increased modestly under drought (ΔeCO_2_ = +42.42 µmol m^−2^ s^−1^) but remained marginal (*p* = 0.07), while *T. triandra* showed an increase under drought (ΔeCO_2_ = +49.05 µmol m^−2^ s^−1^; Table 2) with no response under well‐watered conditions (Table 2). Thus, the contrasting responses of V_cmax_ and V_pmax_ indicate that eCO_2_ did not uniformly amplify the C_4_ carbon‐concentrating pathway but altered the coordination between CO_2_ delivery and Rubisco carboxylation capacity.

Patterns in J_max_ further supported species‐ and water‐dependent upregulation of photosynthetic capacity, consistent with coordinated adjustment of upstream electron transport to match enhanced carboxylation demand. J_max_ was significantly affected by CO_2_ and species, with a pronounced species × water × CO_2_ interaction (Table 1). Under drought, eCO_2_ induced strong upregulation of J_max_ in *H. contortus* (ΔeCO_2_ = +222.63 µmol m^−2^ s^−1^, Table 2), whereas under well‐watered conditions, *M. maximus* upregulated (259.06 µmol m^−2^ s^−1^, Table 2), and in *T. triandra*, no significant J_max_ upregulation was detected (Table 2; Figure 2, Supporting Information Table S2). Thus, upregulation of J_max_ was species‐specific and context dependent, occurring under drought in *H. contortus* but under well‐watered conditions in *M. maximus*.

In contrast to biochemical traits, g_sw_ showed no consistent overall response to eCO_2_ or water availability. The main effect of CO_2_ was marginally non‐significant (Table 1), and neither water availability nor species identity had significant independent effects on g_sw_ (Table 1). However, a significant CO_2_ × water interaction was detected (Table 1), indicating that stomatal responses to eCO_2_ depended on soil water availability. Species‐specific contrasts nevertheless revealed no consistent stomatal adjustment within individual species across water treatments (Table 2; Figure 2). Despite the weak and context‐dependent stomatal responses, iWUE increased significantly under eCO_2_ and differed among species, whereas the main effect of water was not significant (Table 1). Species‐specific contrasts revealed that under drought, iWUE increased significantly in *H. contortus* (ΔeCO_2_ = +90.18 µmol mol^−1^, *p* = 0.021), *M. maximus* (ΔeCO_2_ = +98.29 µmol mol^−1^, Table 2), and T*. triandra* (ΔeCO_2_ = +81.38 µmol mol^−1^, Table 2; Figure 3). Under well‐watered conditions, only *H. contortus* showed a modest but significant increase (ΔeCO_2_ = +82.1 µmol mol^‐1^, Table 2), whereas responses in *M. maximus* and *T. triandra* were not statistically significant. Because g_sw_ remained largely unchanged, increases in iWUE primarily reflected enhanced carbon assimilation rather than stomatal closure.

**Figure 3:**
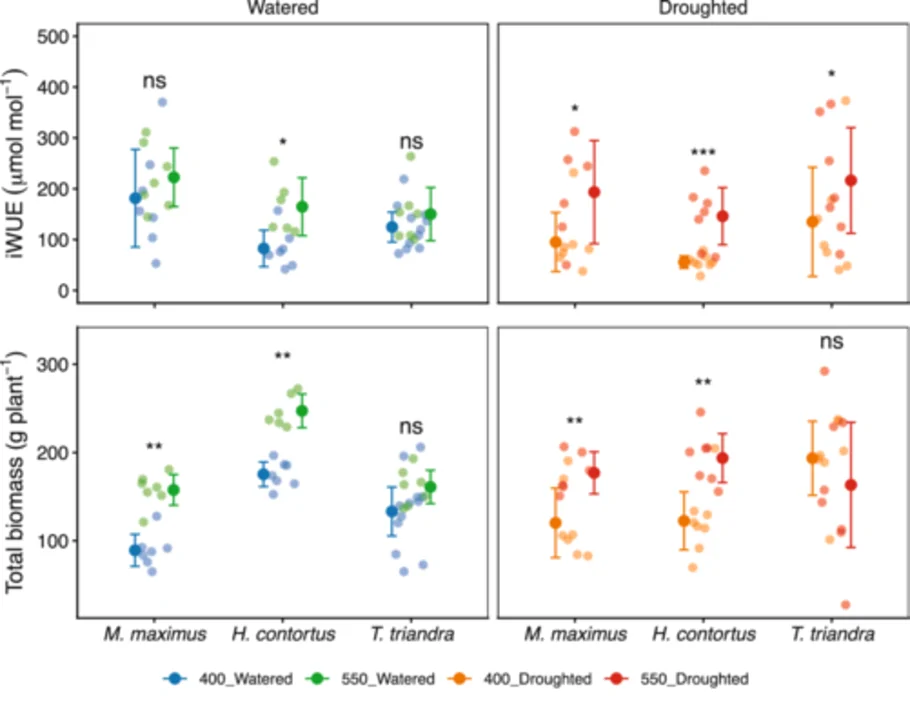
Species‐specific responses of intrinsic water‐use efficiency (iWUE = A/g_sw_) and total biomass to atmospheric CO_2_ concentration under contrasting water availability. Intrinsic water‐use efficiency (iWUE; top row) and total biomass (bottom row) of three C_4_ grass species (*Heteropogon contortus*, *Megathyrsus maximus*, and *Themeda triandra*) grown under watered (left panels) and droughted (right panels) conditions at ambient (400 ppm) and elevated (550 ppm) atmospheric CO_2_. Large points represent means, and error bars indicate 95% confidence intervals (*n* = 7–12 plants per species × treatment combination; exact n values are provided in Supporting Information Table S2). Asterisks indicate the significance of planned within‐species contrasts testing the effect of elevated CO_2_ within each water treatment (ns, not significant; *p* < 0.05; *p* < 0.01; *p* < 0.001). [Color figure can be viewed at wileyonlinelibrary.com]

Total biomass differed significantly among species and exhibited a strong positive response to eCO_2_, with a significant species × CO_2_ interaction (Table 1). Elevated CO_2_ significantly increased total biomass in *H. contortus* and *M. maximus* under both well‐watered and droughted conditions (ΔeCO_2_ = +56.7 to + 71.95 g plant^−1^; all *p* ≤ 0.01; Table 2, Figure 3). In contrast, *T. triandra* showed no significant biomass response under either water treatment, with a non‐significant increase under watered conditions and a non‐significant decrease under drought. Thus, the biomass response to eCO_2_ was species‐specific, with large positive responses concentrated in *H. contortus* and *M. maximus* rather than occurring uniformly across all three C_4_ grasses.

Generally, eCO_2_ enhanced photosynthesis across species, but growth responses were species‐specific and mechanistically distinct. Collectively, these results indicate that eCO_2_ did not drive uniform upregulation of C_4_ pump capacity; instead, biochemical acclimation was expressed more consistently through enhancement of V_cmax_ and, in some cases, J_max_ than through V_pmax_. Stomatal conductance remained largely unchanged across treatments, indicating that biomass responses under eCO_2_ were more closely associated with biochemical acclimation of C_4_ photosynthesis rather than by stomatal water savings alone.

## 4 Discussion

This study investigated the mechanisms underpinning C_4_ grass responses to elevated atmospheric CO_2_ by disentangling stomatal, biochemical, and growth‐level processes across contrasting water regimes. Consistent with long‐standing theoretical expectations, eCO_2_ resulted in modest stimulation of net photosynthesis under well‐watered conditions, but substantially mitigated drought‐induced declines in assimilation, in line with predictions based on near‐saturation of the C_4_ AC_i_ response curve at current atmospheric CO_2_ concentrations (Collatz et al. 1992; Sage and Kubien 2007). In some species, these responses were accompanied by acclimatory upregulation of photosynthetic capacity, indicating that CO_2_ responsiveness in C_4_ grasses cannot be attributed solely to indirect water‐saving effects. Together, the results resolve a key uncertainty highlighted in previous syntheses by demonstrating that sustained exposure to eCO_2_ can induce biochemical acclimation in C_4_ species, although the magnitude and expression of this response depend on physiological context and species traits (Ghannoum et al. 2000; Pignon and Long 2020; Raubenheimer et al. 2023).

Our first hypothesis, that prolonged growth at eCO_2_ increases photosynthetic capacity in C_4_ grasses, was partially supported. Elevated CO_2_ induced significant upregulation of V_cmax_ in *M. maximus* under both well‐watered and droughted conditions, indicating increased Rubisco carboxylation capacity following sustained CO_2_ enrichment. In contrast, *H. contortus* showed only marginal increases in V_cmax_, while *T. triandra* exhibited no change in V_cmax_. These findings align with recent biochemical and modelling analyses suggesting that Rubisco carboxylation capacity, rather than PEPC activity, may represent a primary constraint in C_4_ photosynthesis under contemporary CO_2_ concentrations, and that reallocating resources toward V_cmax_ could enhance carbon gain under eCO_2_ (Pignon and Long 2020; Zhou et al. 2022).

Patterns in J_max_ further support species‐ and context‐dependent acclimatory adjustment of apparent photosynthetic capacity under eCO_2_. J_max_ increased strongly under drought in *H. contortus*, whereas *M. maximus* showed pronounced J_max_ upregulation under well‐watered conditions. In contrast, *T. triandra* showed no significant change in J_max_ under either water regime. These contrasting responses indicate that eCO_2_ did not induce a uniform increase in electron transport capacity across species, but rather stimulated J_max_ only in specific species × water‐treatment contexts. Where present, this upregulation is consistent with coordinated adjustment of electron transport capacity to support increased carboxylation demand and sustained carbon gain. Such coordination between Rubisco activity and RuBP regeneration has been proposed as an important component of sustained photosynthetic enhancement under eCO_2_ in both C_3_ and C_4_ plants (Ghannoum et al. 2000; Leakey et al. 2009). Our results provide evidence consistent with this mechanism in C_4_ grasses, particularly in *H. contortus* under drought and in *M. maximus* under well‐watered conditions.

In contrast to V_cmax_ and J_max_, responses of V_pmax_ were weaker and less consistently affected by water availability. Although V_pmax_ showed a significant overall response to CO_2_, species‐specific contrasts revealed only modest or marginal increases, and these did not align systematically with drought or growth responses. This pattern indicates that eCO_2_ did not uniformly amplify the C_4_ carbon‐concentrating pump. Instead, eCO_2_ altered the coordination between CO_2_ delivery via PEPC and Rubisco carboxylation capacity, with acclimation expressed more consistently on the Rubisco and electron transport sides of the pathway. This finding is consistent with evidence that C_4_ biochemical coordination remains optimised for lower, pre‐industrial CO_2_ concentrations, with limited evolutionary adjustment to rapid anthropogenic CO_2_ increases (Pignon and Long 2020).

Our second hypothesis, which predicted that acclimatory responses would be most pronounced under drought, was partially supported. Across species, eCO_2_ partially offset drought‐induced reductions in net photosynthesis, with the largest stimulation occurring under water limitation. Notably, biochemical enhancement of J_max_ occurred in a species‐ and water‐dependent manner. Under drought conditions, J_max_ increased significantly in *H. contortus*, whereas *M. maximus* exhibited strong upregulation only under well‐watered conditions. Because stomatal conductance showed no consistent decline under eCO_2_, these responses cannot be attributed solely to improved plant water status via reduced transpiration. Instead, drought increased the marginal carbon value of biochemical enhancement, such that additional investment in electron transport and carboxylation capacity yielded proportionally greater returns in carbon assimilation. Under drought, assimilation was otherwise constrained, such that additional investment in electron transport and carboxylation capacity yielded proportionally greater gains in carbon return, thereby favouring coordinated upregulation of photosynthetic components. These findings extend previous frameworks (Wand et al. 1999; Leakey et al. 2009; Stokes et al. 2016) by showing that, in this system, drought amplified not only instantaneous CO_2_ responses but also the expression of acclimatory biochemical adjustments.

The sandy, low‐nutrient soil used in this experiment provides important context for interpreting the observed photosynthetic upregulation. Apparent biochemical capacity traits such as V_cmax_, V_pmax_ and J_max_ require investment in photosynthetic proteins, enzymes and electron transport components, and are therefore potentially sensitive to nutrient availability. Although V_cmax_ is often associated with leaf nitrogen investment (Walker et al. 2014), recent global analyses suggest that this relationship is not universal and that phosphorus availability can also constrain photosynthetic capacity across species and ecosystems (Peng et al. 2021; Ellsworth et al. 2022). In particular, low leaf phosphorus can weaken relationships between photosynthetic capacity and leaf nitrogen, while also constraining V_cmax_ and J_max_ directly or indirectly through effects on photosynthetic biochemistry and electron transport capacity (Ellsworth et al. 2022). Walker et al. (2014) further showed that leaf phosphorus can modify the sensitivity of V_cmax_ to leaf nitrogen, indicating that nitrogen and phosphorus availability may interact in regulating photosynthetic capacity. The fact that some species showed increases in apparent photosynthetic capacity under eCO_2_ despite the use of low‐carbon and low‐nitrogen savanna soil suggests that C_4_ grass responses to eCO_2_ are not necessarily restricted to stomatal water‐saving mechanisms. However, because soil and leaf nutrient availability were not experimentally manipulated or measured in detail, we cannot determine whether nitrogen, phosphorus or their interaction constrained the magnitude of biochemical upregulation or contributed to the species‐specific responses observed here. We therefore interpret these responses as treatment‐associated upregulation of apparent photosynthetic capacity under low‐nutrient savanna soil conditions, rather than as evidence for nutrient‐independent acclimation.

Despite strong increases in intrinsic water‐use efficiency under eCO_2_, stomatal conductance showed weak and context‐dependent responses. Although no consistent overall CO_2_ effect on g_sw_ was detected across species, the significant CO_2_ × water interaction indicates that stomatal responses depended on soil water availability. This apparent decoupling of iWUE and g_sw_ indicates that improved water‐use efficiency arose primarily through enhanced carbon assimilation rather than from consistent reductions in stomatal conductance. Similar patterns have been reported in both wild and cultivated C_4_ species, where eCO_2_ increases iWUE without strong or consistent reductions in stomatal conductance (Wand et al. 1999; Quirk et al. 2019; Raubenheimer et al. 2023). These results caution against attributing iWUE responses solely to stomatal mechanisms and highlight the importance of explicitly separating stomatal and biochemical drivers of CO_2_ responsiveness.

The third hypothesis, which stated that the magnitude and direction of responses differ among species, was also clearly supported. Species differed markedly in their capacity for biochemical acclimation and in the extent to which photosynthetic responses translated into biomass accumulation. Elevated CO_2_ significantly increased total biomass in *H. contortus* and *M. maximus* under both water regimes, whereas *T. triandra* showed no detectable growth response despite increased photosynthesis. Notably, biomass responses aligned more closely with coordinated upregulation of photosynthetic capacity than with instantaneous assimilation alone. In *T. triandra*, CO_2_ stimulation increased net photosynthesis and V_pmax_ under drought but was not accompanied by consistent enhancement of V_cmax_ or J_max_, indicating partial rather than integrated acclimation. This lack of biochemical coordination likely constrained translation of elevated carbon supply into sustained growth. This pattern is consistent with earlier observations from long‐term field experiments, where C_4_ biomass responses to eCO_2_ emerged only after sustained shifts in resource limitation and physiological capacity (Leakey et al. 2009; Pignon and Long 2020).

Collectively, these findings refine the prevailing view that C_4_ responses to eCO_2_ are dominated by indirect effects of water‐saving by demonstrating that, in these species, gains in water‐use efficiency arise primarily from enhanced photosynthesis rather than from stomatal closure. While improved water‐use efficiency remains an important component of C_4_ CO_2_ sensitivity, particularly under drought, our results demonstrate that sustained CO_2_ enrichment can also elicit acclimatory upregulation of photosynthetic capacity in some C_4_ grasses. Importantly, this acclimation is not uniform but instead reflects coordinated shifts in Rubisco carboxylation and electron transport capacity that differ across functional types and environmental contexts. The absence of uniform responses across species highlights the difficulty of generalising C_4_ CO_2_ sensitivity from pathway‐level theory alone and underscores the importance of species‐specific physiological plasticity. Models that assume uniformly minimal biochemical responsiveness of C_4_ photosynthesis to rising CO_2_ may therefore underestimate both the variability and context dependence of C_4_ responses under future climates (Sage and Kubien 2007; Leakey et al. 2009).

A limitation of this study is that plant water status was not measured directly. Leaf water potential, relative water content or hydraulic traits would have provided a more direct assessment of plant‐level drought stress and would have strengthened interpretation of the CO_2_ × water interaction. Our inference of water limitation is therefore based on continuous soil VWC measurements and the associated soil water retention relationship, which showed that droughted pots remained within a soil moisture range corresponding to strongly negative soil water potentials for most of the experiment. These soil‐based estimates indicate prolonged exposure to low soil water availability, but they should not be interpreted as direct measurements of plant hydraulic status. Future experiments should combine continuous soil moisture monitoring with direct measurements of plant water status to determine how eCO_2_ affects plant hydraulic status and its relationship with photosynthetic capacity.

A further limitation is that AC_i_ curves were measured during a single late‐season campaign, and repeated independent spot measurements of gas exchange at growth CO_2_ were not conducted under naturally varying chamber conditions. However, the measurements were made after prolonged exposure to the CO_2_ and water treatments and therefore capture treatment‐associated differences in apparent photosynthetic capacity at an ecologically relevant stage of plant development. The alignment between these physiological responses and final biomass accumulation indicates that the observed differences were not isolated leaf‐level responses, but were associated with integrated whole‐plant performance. Future studies incorporating repeated AC_i_ curves and spot gas‐exchange measurements under growth conditions would help determine the temporal stability of these responses across the growing season.

In conclusion, this study demonstrates that C_4_ grass responses to eCO_2_ extend beyond indirect effects mediated by water relations and can include acclimatory upregulation of photosynthetic capacity, particularly under water limitation. By linking biochemical, physiological, and growth responses across species, our findings highlight the importance of physiological plasticity in shaping C_4_ productivity under rising atmospheric CO_2_ and increasing drought frequency, with implications for predicting grassland responses under future climate scenarios.

## Supporting information


Supporting File 1

Supporting File 2

## Data Availability

The data that support the findings of this study are available from the corresponding author upon reasonable request.
