# Supplementary material for: CO2 Fertilisation of Growth in Wild C4 Grasses may be Amplified by Upregulated Photosynthesis
Source: Plant Cell Environ. 2026 Aug 3;49(11):8870–82. doi: 10.1111/pce.70785 (PMC13646657; doi:10.1111/pce.70785)
Supplement: Supplementary file 1 [file PCE-49-8870-s001.pdf]

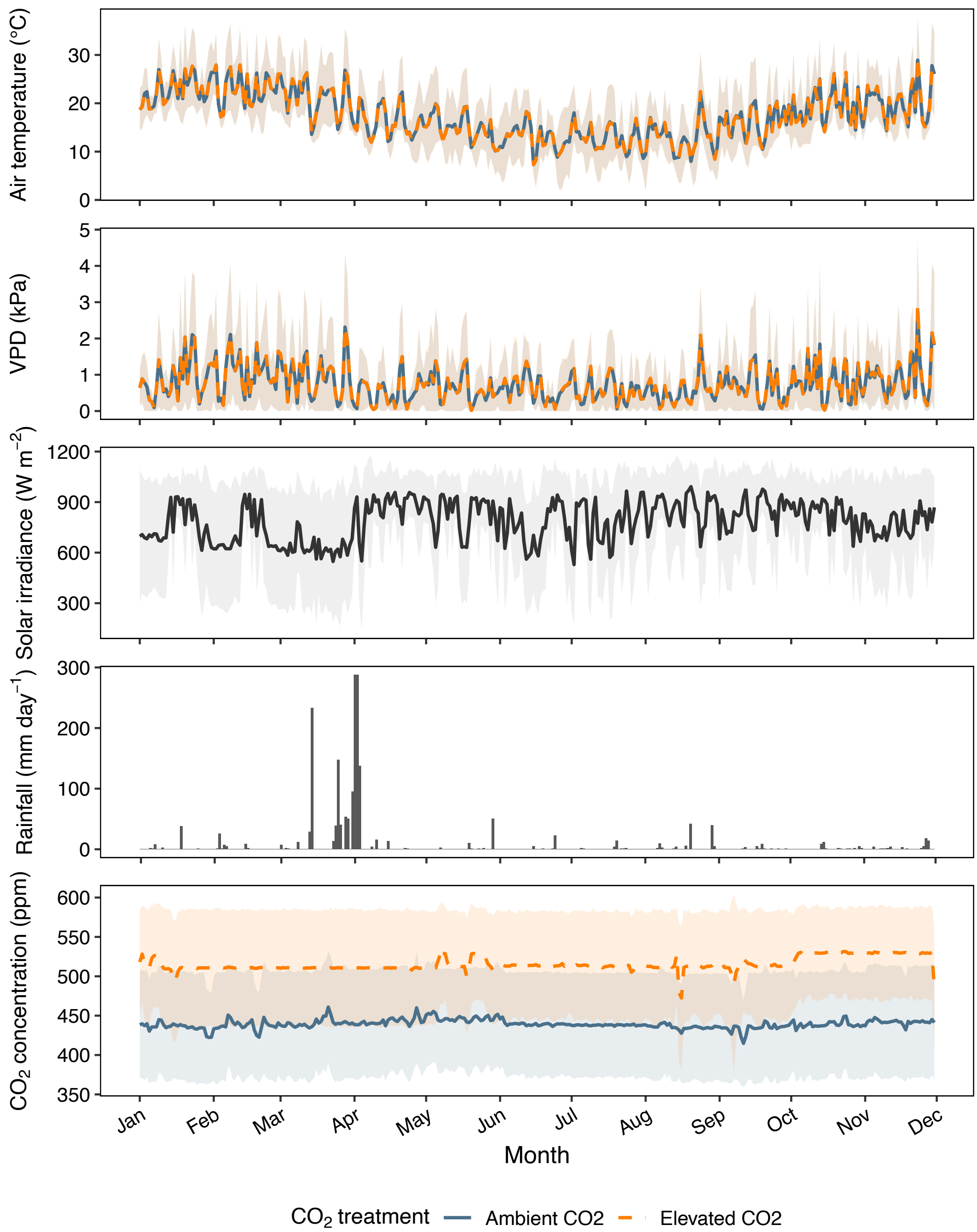

Figure S1. Environmental conditions during the 2022 experimental period. Daily mean air temperature, vapour pressure deficit (VPD), solar irradiance, rainfall and chamber CO_2 concentration are shown across the experimental period. Air temperature, VPD and CO_2 concentration were summarised separately for chambers assigned to ambient/reference CO_2 and elevated CO_2 treatments. Chamber CO_2 concentration represents daily means from chambers assigned to ambient/reference CO_2 and elevated CO_2 treatments. Target concentrations were 400 ppm and 550 ppm, respectively, but realised chamber concentrations varied through time due to normal OTC operation and environmental conditions. VPD was calculated from chamber air temperature and relative humidity. Solar irradiance and rainfall were recorded at facility level and are therefore shown as common environmental conditions across all chambers. Lines represent daily means, shaded ribbons indicate ± SD for chamber-level variables, and rainfall is shown as daily totals.

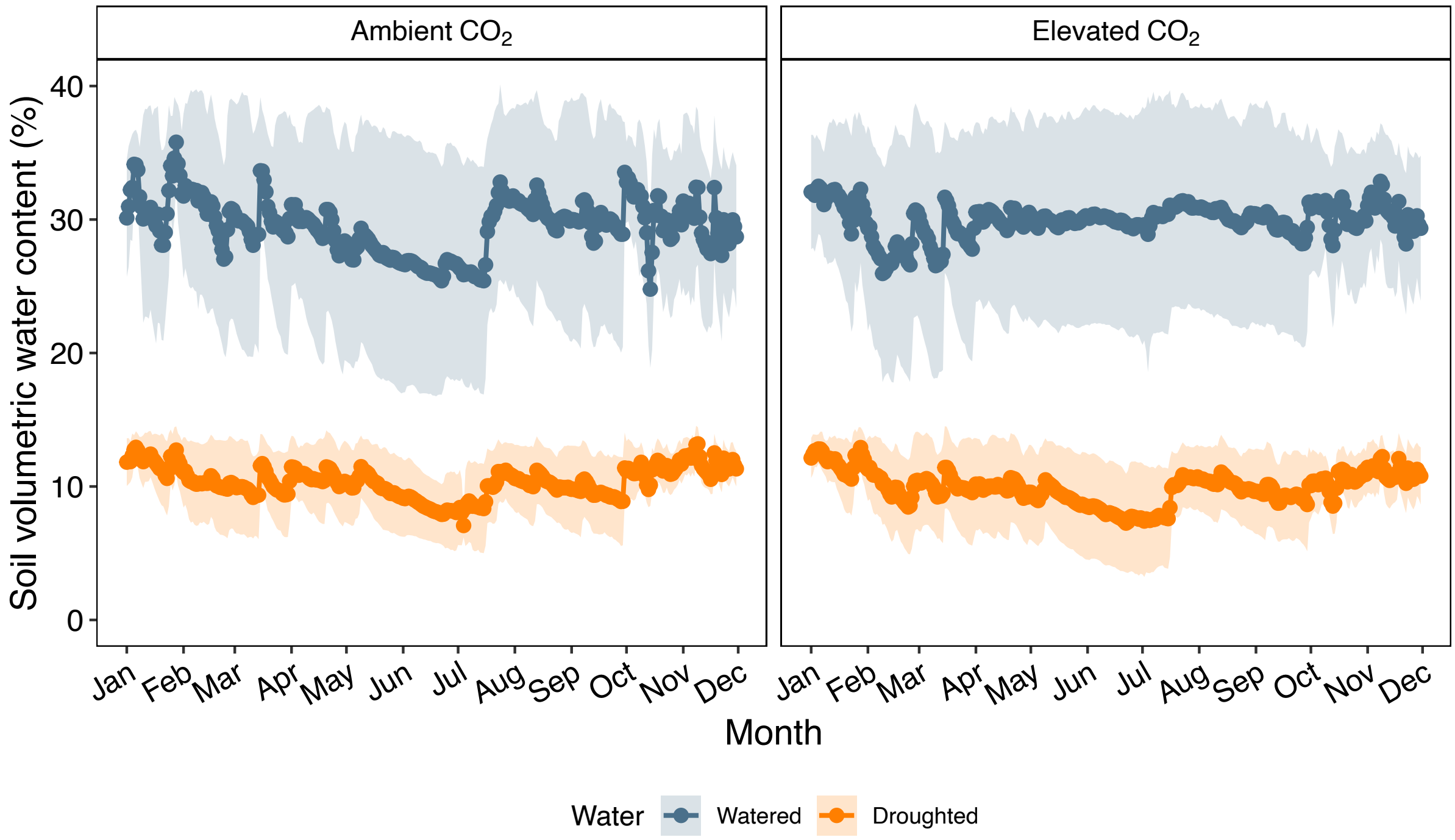

Figure S2. Seasonal variation in soil volumetric water content (VWC) across watered and droughted treatments under ambient/reference and elevated CO_2. Lines represent daily mean VWC (%) for each water treatment, while shaded ribbons indicate variation around the mean. Blue represents watered pots and orange represents droughted pots. Panels show ambient/reference CO_2 and elevated CO_2 treatments separately.

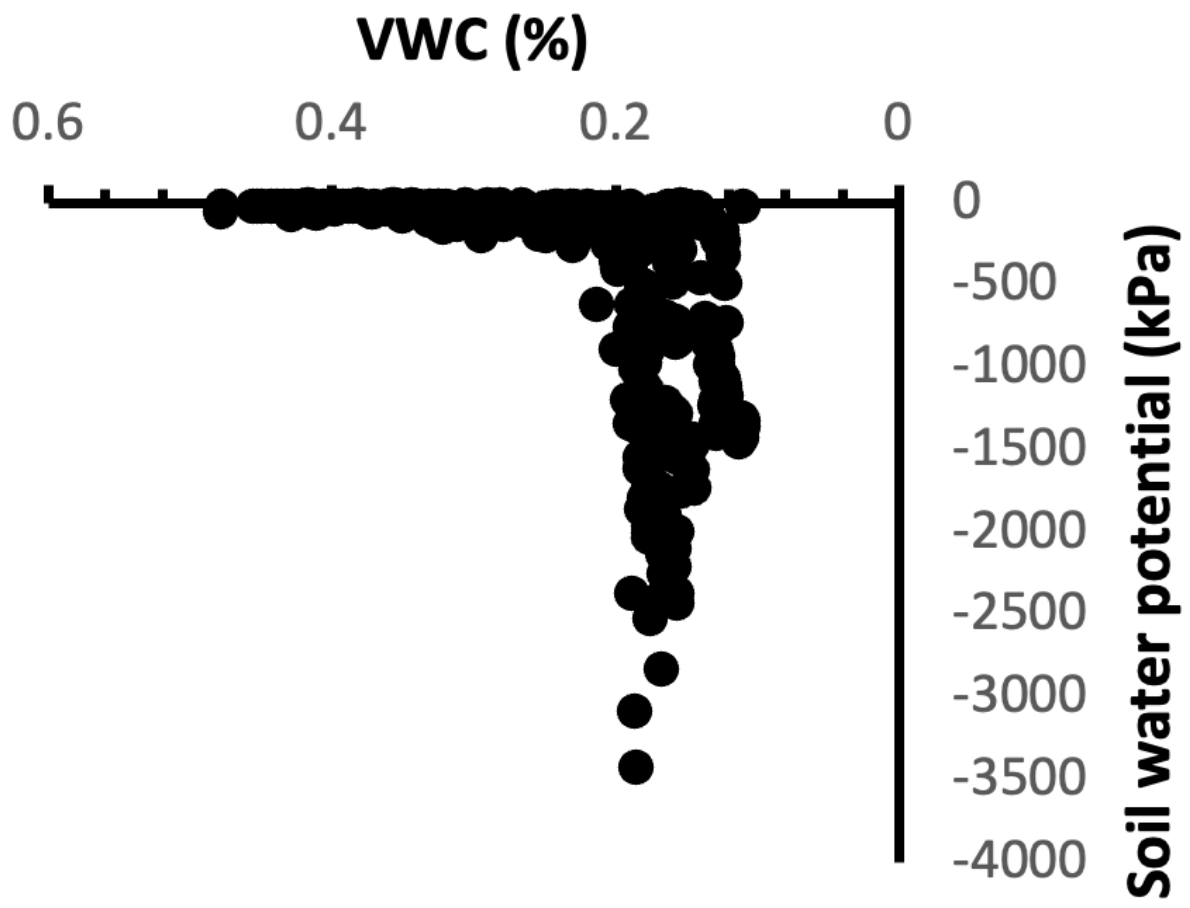

Figure S3. Relationship between volumetric soil water content (VWC) and soil water potential for soils used in the grass experiment. Soil water potential declined non-linearly with decreasing VWC, with a sharp decline occurring over a relatively narrow range of soil moisture. The scatter in VWC values associated with a given soil water potential reflects measurements taken during both soil drying and re-wetting phases, producing a characteristic hysteresis in the soil water retention relationship.

Supplementary Methods S1. Calculation of stomatal and non-stomatal limitations

Stomatal and non-stomatal limitations to photosynthesis were calculated from predicted assimilation values derived from fitted AC_i curves. For each fitted curve, predicted assimilation was extracted at the growth atmospheric CO_2 concentration ($ApredC_a$) and at the corresponding intercellular CO_2 concentration ($ApredC_i$). Stomatal limitation was calculated as the proportional reduction in predicted assimilation associated with the drawdown from C_a to C_i:

$$\mathrm{SL} = \left[\frac{(\mathrm{ApredCa} - \mathrm{ApredCi})}{\mathrm{ApredCa}}\right] X\ 100$$

This calculation was performed separately for watered and droughted plants. To compare relative stomatal and non-stomatal limitations under drought, watered and droughted curves were paired within each species × CO_2 treatment combination. In this paired framework, W represents predicted assimilation at C_a under watered conditions, X represents predicted assimilation at C_i under watered conditions, Y represents predicted assimilation at C_a under droughted conditions, and Z represents predicted assimilation at C_i under droughted conditions.

Stomatal limitation under watered conditions was calculated as $[(W - X) / W] \times 100$, while stomatal limitation under droughted conditions was calculated as $[(Y - Z) / Y] \times 100$. Relative stomatal limitation under drought was calculated as $[(Y - Z) / X] \times 100$, and relative metabolic limitation was calculated as $[(X - Z) / (Y - Z)] \times 100$. Relative metabolic limitation values were constrained to the biologically interpretable range of 0–100%. Because these calculations were based on AC_i curve predictions, they should be interpreted as limitation indices under the standardised cuvette conditions used for gas-exchange measurements, rather than as direct estimates of realised limitation under naturally fluctuating chamber conditions.

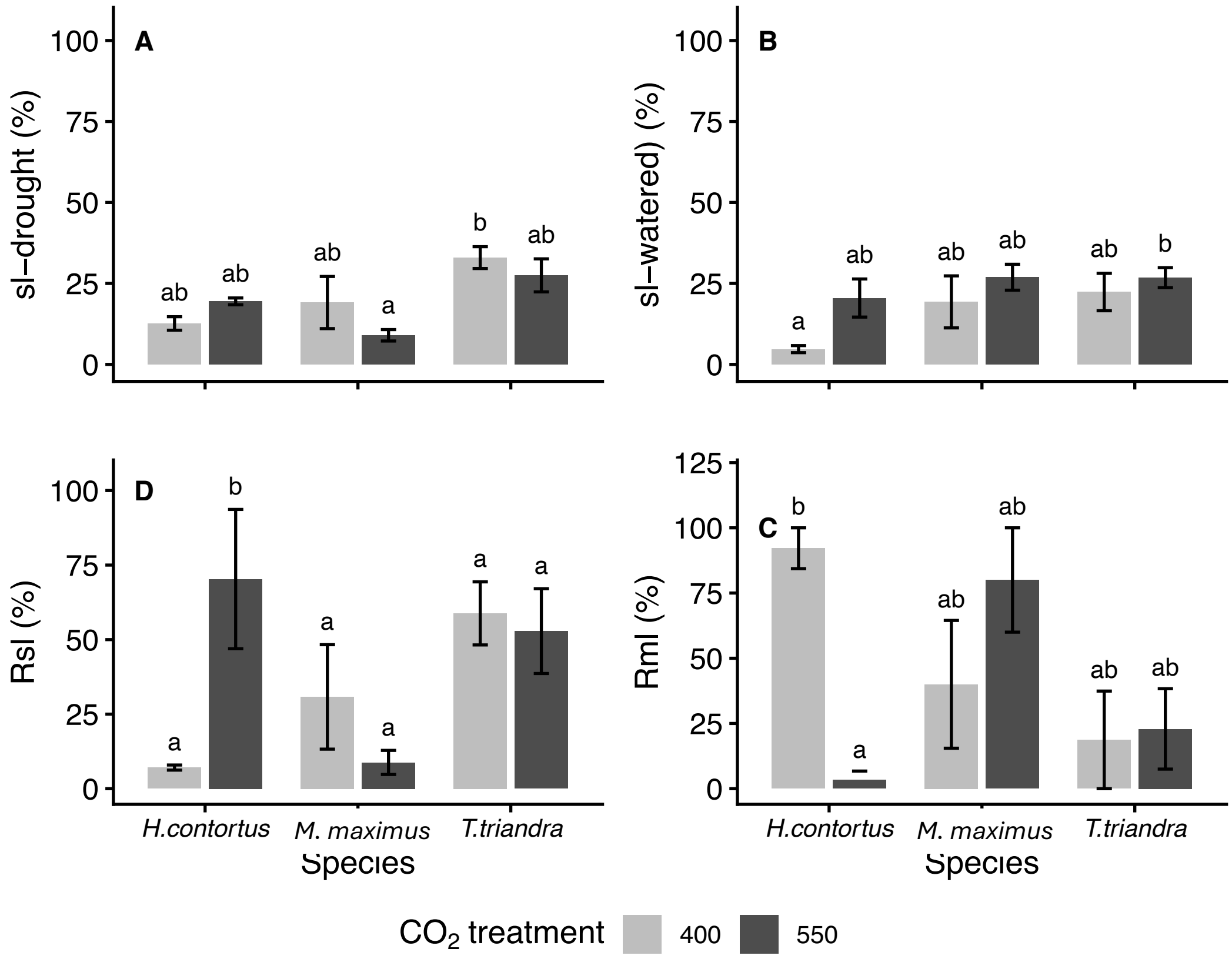

Figure S4. Stomatal and non-stomatal limitations to photosynthesis in three grass species (*Heteropogon contortus*, *Megathyrsus maximus*, and *Themeda triandra*) grown under ambient (400 ppm) and elevated (550 ppm) CO_2. Values were calculated from predicted assimilation derived from fitted AC_i curves measured under standardised cuvette conditions. Stomatal limitation was calculated as the proportional reduction in predicted assimilation caused by the drawdown from atmospheric CO_2 concentration (Ca) to intercellular CO_2 concentration (C_i). Panels show stomatal limitation under droughted conditions, stomatal limitation under watered conditions, relative stomatal limitation and relative metabolic limitation. Bars represent means ± SE (n = 7–12 plants per species × treatment combination; exact n values are provided in Table S2). Different lowercase letters indicate significant differences among species × CO_2 treatment combinations.
