# Supplementary material for: CO2 Fertilisation of Growth in Wild C4 Grasses may be Amplified by Upregulated Photosynthesis
Source: Plant Cell Environ. 2026 Aug 3;49(11):8870–82. doi: 10.1111/pce.70785 (PMC13646657; doi:10.1111/pce.70785)
Supplement: Supplementary file 2 [file PCE-49-8870-s002.docx]

**Supplementary Tables**

**Table S_1_.** Duration and severity of soil water limitation across CO_2_ and water treatments. Values are based on daily mean volumetric water content (VWC) recorded over 334 days in pots assigned to watered and droughted treatments under ambient and elevated CO_2_. The number and percentage of days below selected VWC thresholds are shown to quantify the duration of low soil water availability. Thresholds are interpreted with reference to the soil water retention relationship shown in Fig. S3, where soil water potential declined sharply at low VWC.

| Water | CO_2_ | Monitoring period (days) | Mean VWC (%) | VWC range (%) | Days <18% VWC | Days <15% VWC | Days <12% VWC | Days <10% VWC |
| --- | --- | --- | --- | --- | --- | --- | --- | --- |
| Watered | Ambient | 334 | 29.51 ± 2.07 | 24.78–35.79 | 0 (0%) | 0 (0%) | 0 (0%) | 0 (0%) |
| Watered | Elevated | 334 | 29.94 ± 1.24 | 25.96–32.86 | 0 (0%) | 0 (0%) | 0 (0%) | 0 (0%) |
| Droughted | Ambient | 334 | 10.36 ± 1.15 | 7.08–13.19 | 334 (100%) | 334 (100%) | 307 (91.9%) | 127 (38.0%) |
| Droughted | Elevated | 334 | 9.93 ± 1.22 | 7.25–12.92 | 334 (100%) | 334 (100%) | 314 (94.0%) | 163 (48.8%) |

**Table S_2_.** Raw percentage changes in physiological and biomass traits under elevated CO_2_. Raw treatment mean values are shown for plants grown under ambient/reference CO_2_ (400 ppm) and elevated CO_2_ (550 ppm), together with sample sizes, absolute changes and percentage changes under elevated CO_2_ for each species × water treatment combination. Percentage change was calculated as [(mean at 550 ppm − mean at 400 ppm) / mean at 400 ppm] × 100. Positive values indicate higher trait values under elevated CO_2_, whereas negative values indicate lower values under elevated CO_2_. The table also includes J_max_/V_cmax_ and A/V_cmax_ ratios to evaluate whether electron transport capacity, carboxylation capacity and realised photosynthesis changed proportionally across treatments.

| Species | Water | Variable | n at 400 ppm | n at 550 ppm | Mean at 400 ppm | Mean at 550 ppm | SD at 400 ppm | SD at 550 ppm | SE at 400 ppm | SE at 550 ppm | Absolute change under eCO_2_ | % change under eCO_2_ |
| --- | --- | --- | --- | --- | --- | --- | --- | --- | --- | --- | --- | --- |
| *H. contortus* | Droughted | A | 8 | 7 | 13.2 | 23.34 | 2.6 | 2.58 | 0.92 | 0.98 | 10.1 | 76.72 |
| *H. contortus* | Watered | A | 7 | 7 | 16.64 | 25.03 | 2.8 | 3.95 | 1.06 | 1.49 | 8.39 | 50.43 |
| *M. maximus* | Droughted | A | 7 | 7 | 10.17 | 27.55 | 2.16 | 1.12 | 0.82 | 0.42 | 17.4 | 170.9 |
| *M. maximus* | Watered | A | 7 | 7 | 22.15 | 29 | 2.81 | 1.5 | 1.06 | 0.57 | 6.85 | 30.94 |
| *T. triandra* | Droughted | A | 7 | 8 | 18.04 | 24.3 | 3.94 | 2.03 | 1.49 | 0.72 | 6.26 | 34.69 |
| *T. triandra* | Watered | A | 12 | 7 | 21.02 | 23.9 | 2.29 | 3.65 | 0.66 | 1.38 | 2.88 | 13.71 |
| *H. contortus* | Droughted | A/V_cmax_ | 8 | 7 | 0.56 | 0.34 | 0.46 | 0.13 | 0.16 | 0.05 | -0.2 | -39.19 |
| *H. contortus* | Watered | A/V_cmax_ | 7 | 7 | 0.41 | 0.44 | 0.17 | 0.37 | 0.06 | 0.14 | 0.04 | 9 |
| *M. maximus* | Droughted | A/V_cmax_ | 7 | 7 | 0.28 | 0.48 | 0.08 | 0.29 | 0.03 | 0.11 | 0.19 | 69.09 |
| *M. maximus* | Watered | A/V_cmax_ | 7 | 7 | 0.55 | 0.29 | 0.27 | 0.09 | 0.1 | 0.03 | -0.3 | -47.57 |
| *T. triandra* | Droughted | A/V_cmax_ | 7 | 8 | 0.31 | 0.38 | 0.08 | 0.22 | 0.03 | 0.08 | 0.07 | 23.39 |
| *T. triandra* | Watered | A/V_cmax_ | 7 | 7 | 0.3 | 0.32 | 0.08 | 0.08 | 0.03 | 0.03 | 0.03 | 9.36 |
| *H. contortus* | Droughted | J_max_ | 8 | 7 | 112.28 | 341.38 | 42.9 | 128.6 | 15.2 | 48.6 | 229 | 204.1 |
| *H. contortus* | Watered | J_max_ | 7 | 7 | 191.32 | 228.81 | 36.4 | 53.4 | 13.7 | 20.2 | 37.5 | 19.6 |
| *M. maximus* | Droughted | J_max_ | 7 | 7 | 287.28 | 266.03 | 216 | 89.94 | 81.8 | 34 | -21 | -7.4 |
| *M. maximus* | Watered | J_max_ | 7 | 7 | 175.62 | 405.33 | 74.7 | 85.34 | 28.2 | 32.3 | 230 | 130.8 |
| *T. triandra* | Droughted | J_max_ | 7 | 8 | 261.53 | 304.91 | 63.6 | 87.71 | 24 | 31 | 43.4 | 16.59 |
| *T. triandra* | Watered | J_max_ | 7 | 7 | 264.39 | 302.27 | 158 | 32.98 | 59.7 | 12.5 | 37.9 | 14.33 |
| *H. contortus* | Droughted | J_max_/V_cmax_ | 8 | 7 | 4.05 | 4.93 | 1.64 | 2.82 | 0.58 | 1.07 | 0.88 | 21.81 |
| *H. contortus* | Watered | J_max_/V_cmax_ | 7 | 7 | 4.46 | 3.33 | 1.1 | 1.03 | 0.41 | 0.39 | -1.1 | -25.51 |
| *M. maximus* | Droughted | J_max_/V_cmax_ | 7 | 7 | 7.31 | 3.86 | 4.98 | 1.61 | 1.88 | 0.61 | -3.5 | -47.17 |
| *M. maximus* | Watered | J_max_/V_cmax_ | 7 | 7 | 3.7 | 3.87 | 1.3 | 1.04 | 0.49 | 0.39 | 0.17 | 4.64 |
| *T. triandra* | Droughted | J_max_/V_cmax_ | 7 | 8 | 4.47 | 4.24 | 1.46 | 0.85 | 0.55 | 0.3 | -0.2 | -5.16 |
| *T. triandra* | Watered | J_max_/V_cmax_ | 7 | 7 | 3.43 | 4.12 | 1.43 | 1.1 | 0.54 | 0.42 | 0.7 | 20.28 |
| *H. contortus* | Droughted | Total biomass | 8 | 7 | 122.54 | 193.76 | 39.3 | 29.94 | 13.9 | 11.3 | 71.2 | 58.12 |
| *H. contortus* | Watered | Total biomass | 7 | 7 | 175.34 | 253.75 | 15.1 | 23.78 | 5.69 | 8.99 | 78.4 | 44.71 |
| *M. maximus* | Droughted | Total biomass | 7 | 7 | 120.26 | 170.65 | 42.5 | 26.56 | 16.1 | 10 | 50.4 | 41.9 |
| *M. maximus* | Watered | Total biomass | 7 | 7 | 89.21 | 157.67 | 19.6 | 18.75 | 7.4 | 7.09 | 68.5 | 76.74 |
| *T. triandra* | Droughted | Total biomass | 7 | 8 | 193.54 | 163.29 | 45.4 | 84.76 | 17.1 | 30 | -30 | -15.63 |
| *T. triandra* | Watered | Total biomass | 12 | 7 | 133.22 | 161 | 43.6 | 20.42 | 12.6 | 7.72 | 27.8 | 20.85 |
| *H. contortus* | Droughted | V_cmax_ | 8 | 7 | 48.41 | 78.09 | 62 | 31.13 | 21.9 | 11.8 | 29.7 | 61.3 |
| *H. contortus* | Watered | V_cmax_ | 7 | 7 | 47.48 | 82.26 | 23.3 | 53.44 | 8.8 | 20.2 | 34.8 | 73.26 |
| *M. maximus* | Droughted | V_cmax_ | 7 | 7 | 39.2 | 81.65 | 14 | 51.35 | 5.29 | 19.4 | 42.5 | 108.3 |
| *M. maximus* | Watered | V_cmax_ | 7 | 7 | 50.08 | 112.49 | 24.7 | 44.91 | 9.34 | 17 | 62.4 | 124.6 |
| *T. triandra* | Droughted | V_cmax_ | 7 | 8 | 63.79 | 73.12 | 27.7 | 20.78 | 10.5 | 7.35 | 9.32 | 14.62 |
| *T. triandra* | Watered | V_cmax_ | 7 | 7 | 77.4 | 77.55 | 23.5 | 21.63 | 8.88 | 8.17 | 0.15 | 0.2 |
| *H. contortus* | Droughted | V_pmax_ | 8 | 7 | 50.59 | 93.01 | 61.2 | 50.76 | 21.6 | 19.2 | 42.4 | 83.85 |
| *H. contortus* | Watered | V_pmax_ | 7 | 7 | 45.85 | 82.18 | 10.5 | 66.45 | 3.95 | 25.1 | 36.3 | 79.23 |
| *M. maximus* | Droughted | V_pmax_ | 7 | 7 | 39.06 | 101.67 | 15 | 63.18 | 5.66 | 23.9 | 62.6 | 160.3 |
| *M. maximus* | Watered | V_pmax_ | 7 | 7 | 38.08 | 151.45 | 10.3 | 51.98 | 3.91 | 19.7 | 113 | 297.7 |
| *T. triandra* | Droughted | V_pmax_ | 7 | 8 | 71.2 | 120.25 | 26.6 | 71.77 | 10.1 | 25.4 | 49.1 | 68.89 |
| *T. triandra* | Watered | V_pmax_ | 7 | 7 | 38.33 | 59.96 | 16.6 | 11.25 | 6.26 | 4.25 | 21.6 | 56.44 |
| *H. contortus* | Droughted | g_sw_ | 8 | 7 | 0.25 | 0.19 | 0.08 | 0.08 | 0.03 | 0.03 | -0.1 | -24.95 |
| *H. contortus* | Watered | g_sw_ | 7 | 7 | 0.23 | 0.15 | 0.09 | 0.06 | 0.03 | 0.02 | -0.1 | -35.4 |
| *M. maximus* | Droughted | g_sw_ | 7 | 7 | 0.13 | 0.18 | 0.05 | 0.16 | 0.02 | 0.06 | 0.05 | 37.36 |
| *M. maximus* | Watered | g_sw_ | 7 | 7 | 0.17 | 0.14 | 0.1 | 0.03 | 0.04 | 0.01 | -0 | -16.38 |
| *T. triandra* | Droughted | g_sw_ | 7 | 7 | 0.22 | 0.15 | 0.14 | 0.1 | 0.05 | 0.04 | -0.1 | -32.47 |
| *T. triandra* | Watered | gsw | 11 | 7 | 0.19 | 0.17 | 0.07 | 0.05 | 0.02 | 0.02 | -0 | -7.7 |
| *H. contortus* | Droughted | iWUE | 8 | 7 | 55.8 | 145.99 | 14.5 | 60.72 | 5.12 | 23 | 90.2 | 161.6 |
| *H. contortus* | Watered | iWUE | 7 | 7 | 82.54 | 204.8 | 38.6 | 117.2 | 14.6 | 44.3 | 122 | 148.1 |
| *M. maximus* | Droughted | iWUE | 7 | 7 | 95.1 | 270.7 | 62.7 | 222.7 | 23.7 | 84.2 | 176 | 184.6 |
| *M. maximus* | Watered | iWUE | 7 | 7 | 181.35 | 222.48 | 104 | 62.38 | 39.3 | 23.6 | 41.1 | 22.68 |
| *T. triandra* | Droughted | iWUE | 7 | 7 | 134.97 | 216.35 | 116 | 112.4 | 43.9 | 42.5 | 81.4 | 60.29 |
| *T. triandra* | Watered | iWUE | 11 | 7 | 124.75 | 169.53 | 43.8 | 59.8 | 13.2 | 22.6 | 44.8 | 35.9 |
